# ASO Author Reflections: Need for a Textbook Outcome in Gastric Surgery (TOGS) Specific for Gastric Cancer

**DOI:** 10.1245/s10434-026-19645-7

**Published:** 2026-04-14

**Authors:** Ludovico Carbone, Yo-Seok Cho, Hyuk-Joon Lee, Seong-Ho Kong, Do Joong Park, Daniele Marrelli, Franco Roviello

**Affiliations:** 1 Division of Gastrointestinal Surgery, Department of Surgery, Seoul National University Hospital, Seoul, Republic of Korea; 2 Department of Medicine Surgery and Neuroscience, University of Siena, Siena, Italy; 3 Department of Surgery, Seoul National University College of Medicine (SNUCM), Seoul, Republic of Korea; 4 Cancer Research Institute, Seoul National University College of Medicine (SNUCM), Seoul, Republic of Korea; 5 Department of Oncology, Azienda Ospedaliero Universitaria Senese (AOUS), Siena, Italy

## Past

Ensuring high-quality care for patients with cancer has become a global priority. Centralization of complex surgical procedures has been consistently associated with lower morbidity and mortality. However, mortality rates after major resection for gastric cancer still range from 2% to 10%, even in high-volume institutions, and no consensus exists on how to define volume cutoff.

Textbook Outcome has recently emerged as a holistic indicator to assess surgical quality in a more comprehensive way.^1^ Although the Dutch group first proposed a definition for gastrointestinal cancer, its reproducibility has been inconsistent across studies.^2^

## Present

To enable more reliable comparisons of perioperative outcomes across specialised centres, the Italian Research Group for Gastric Cancer (GIRCG) developed the Textbook Outcome in Gastric Surgery (TOGS), specifically designed for gastric cancer.^3^

TOGS includes eight criteria:

- no intraoperative complications;
- negative resection margins at pathological examination;
- adequate lymphadenectomy (more than 20 nodes removed in subtotal gastrectomy and more than 25 in total gastrectomy);
- no reintervention within 30 days after surgery;
- no unplanned intensive care unit admission within 30 days;
- no unplanned hospital readmission within 90 days;
- no mortality within 90 days;
- tumour board evaluation, when indicated by local guidelines/reccomendations (e.g., timely initiation and compliance with neoadjuvant therapy).

Each of these metrics must be met to achieve TOGS.

Earlier results have demonstrated that TOGS correlates with reduced mortality and shorter hospital stay, indipendent of adjustment for age, BMI and pathological stages in 1988 Western patients.^3^

Our study is the first external validation of TOGS in an East Asian population, based on 5806 patients who underwent curative gastrectomy at Seoul National University Hospital in South Korea. Two factors emerged as particularly important in determining TOGS achievement. First, adequate lymph node retrieval, the criterion most directly linked to surgical quality, was met in 93.7% of patients and was the single parameter that most closely mirrored overall TOGS success over the study period. Importantly, nodal metastasis status (pN) was not an independent predictor of TOGS failure, confirming that it is the completeness of lymphadenectomy, not nodal involvement per se, that drives better outcome achievement. Second, the growing use of minimally invasive surgery played a key role: the rate of laparoscopic and robotic procedures increased from 68.5% before 2018 to > 90% from 2022 onward, and was independently associated with a 1.51 higher probability of achieving TOGS. Finally, beyond perioperative outcomes, TOGS achievement was strongly associated with improved 5-year overall survival (*P* <0.001): 87.6% versus 75.6% overall, 94.9% versus 89.3% in stage I, 88.0% versus 74.6% in stage II, and 59.0% versus 46.6% in stage III, showing that achieving high surgical quality has a real and measurable impact on oncological outcomes, regardless of disease stage.^4^

## Future

These results support TOGS as a promising international benchmark for gastric cancer surgery. Its strength lies in capturing multiple dimensions of surgical quality within a single metric, going beyond traditional indicators such as mortality or complication rates alone. Unlike volume-based thresholds, which are arbitrarily defined and difficult to generalise, TOGS reflects the overall quality of the perioperative process and has demonstrated a consistent association with long-term survival.

In response to the question raised by UK colleagues as to whether a composite outcome represents a useful metric or merely a moving target, one might paraphrase the British writer John Ruskin “Quality is never an accident, it is always the result of high intention, sincere effort, intelligent direction and skillful execution.”^2^

Further validation of this version of TOGS is being actively promoted in East and West centres, with the goal of confirming its applicability across different healthcare settings and refining its criteria where needed.^5^ If these efforts confirm the present findings, TOGS has the potential to become a shared standard for benchmarking and improving surgical quality in gastric cancer worldwide.
